# Multiplying insights from perturbation experiments: predicting new perturbation combinations

**DOI:** 10.15252/msb.202311667

**Published:** 2023-05-11

**Authors:** Joshua Welch

**Affiliations:** ^1^ University of Michigan Ann Arbor MI USA

**Keywords:** Computational Biology, Methods & Resources

## Abstract

Experimentally exploring the effect of all perturbation combinations is not feasible. In their recent study, Theis and colleagues (Lotfollahi *et al*, 2023) present an approach that uses deep generative models to predict the effects of new perturbations from high‐throughput single perturbation experiments.

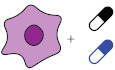

Recent advances in multiplexed single‐cell transcriptomics have allowed generating vast amounts of drug and genetic perturbation data. Experimentally exploring the combinatorial perturbation space is not feasible, emphasizing the need for computational methods that could predict the effect of perturbations. In their recent study, Theis and colleagues (Lotfollahi *et al*, 2023) present a new approach that uses deep generative models to predict the effects of new perturbations (such as drug or gene combinations that have not been experimentally measured) from high‐throughput single perturbation experiments.

Advances in experimental technologies have made high‐throughput single‐cell perturbation measurements increasingly easy to obtain. Activating, repressing, or editing specific genomic targets with CRISPR‐based technologies allows focused genetic perturbations, and the effects of hundreds or thousands of perturbations on gene expression can be measured using Perturb‐Seq (Dixit *et al*, 2016) or CROP‐seq (Datlinger *et al*, 2017). Similarly, with techniques such as Sci‐Plex (Srivatsan *et al*, 2020), it is now possible to measure gene expression after drug treatment, at single‐cell resolution, across many different cell types. The cellular perturbation response is highly complex because perturbations can both directly affect gene targets and indirectly cause changes that propagate through gene regulatory interactions. Thus, understanding the consequences of perturbations requires considering the entire high‐dimensional molecular state of a cell. This makes single‐cell perturbation data a remarkable new resource for deciphering perturbation responses. However, the space of perturbation effects is far too large to measure exhaustively. For example, the number of possible drug‐like molecules is estimated to exceed 10^60^. Similarly, there are more than 200 million combinations of single‐ and double‐gene perturbations alone. The combinatorial explosion becomes more pronounced when considering multiple drug doses or multiple guide RNAs, multiple cell types, and multiple drug or gene combinations.

In a seemingly unrelated trend, machine learning approaches for generating novel examples of high‐dimensional data have rapidly advanced. In particular, using neural networks to train deep generative models has proven remarkably effective at generating text and image data. For example, deep generative models can draw pictures of new human faces that look like real people, even though no such person exists (Karras *et al*, 2019). Similar techniques have proven effective at generating new sentences and paragraphs that read just like prose from a human author (preprint: OpenAI, 2023). A particularly powerful property behind the success of these deep generative models is their ability to disentangle complex factors of variation in the training data. For example, a deep generative model trained on pictures of chairs can learn the effects of color, fabric type, and chair style on image pixel values. Disentangling such factors allows a remarkable result: Deep generative models can generalize beyond the training data by combining the underlying factors in ways not previously observed. Thus, we can combine concepts such as “armchair,” “red,” “avocado,” and “gummy bear” in different ways to draw pictures of imaginary armchairs in the shape of an avocado or gummy bear (Fig 1A).

**Figure 1:**
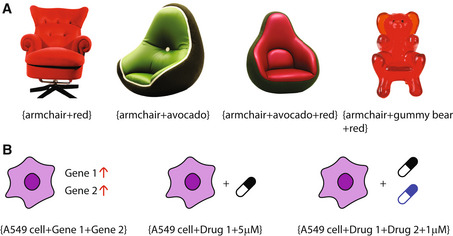
(A) Deep generative models can combine concepts such as “armchair,” “red,” “avocado,” and “gummy bear” in new ways to draw pictures of imaginary armchairs in the shape of an avocado or gummy bear. Images generated by DALL‐E2. (B) Similarly, CPA can predict gene expression profiles for cells with unobserved gene combinations, drug dosages, or drug combinations based on the expression profiles obtained from single perturbations.

In their recent work, Lotfollahi *et al* (2023) combine these two strands of research (single‐cell perturbation measurements and deep generative models) to predict the effects of new perturbation combinations that have not yet been experimentally analyzed, such as drug dosages, cell types, or time points. The key contribution of the study is a deep generative model called compositional perturbation autoencoder (CPA). Similar to how deep generative models can draw pictures with combinations of attributes not seen in the training data, CPA can generate gene expression profiles of cells for new combinations of perturbations (Fig 1B). CPA is trained on large perturbation datasets from experiments such as Perturb‐seq, CROP‐seq, or sci‐Plex. In an extensive set of evaluations, the authors showed that CPA can predict the effects of new gene activation combinations, new drug doses, and even new drug combinations. This effectively “multiplies” the utility of existing single‐cell perturbation datasets, as the authors demonstrated by predicting gene expression profiles for the additional 98% of all possible two‐gene combinations that were not measured in a Perturb‐Seq dataset. In an impressive proof of principle, they used CPA to predict the gene expression profiles induced by unseen drug combinations. They then experimentally validated these results by creating a new single‐cell dataset, which demonstrated the accuracy of the model predictions. While CPA is designed to predict new combinations of perturbations already seen in the training data, an extension of the model (called ChemCPA) uses molecule embeddings to predict unseen single‐drug treatments.

CPA opens several exciting future directions. First, while CPA makes predictions using neural networks rather than a directly interpretable model, these large‐scale predictions provide an opportunity to elucidate mechanisms of gene regulation. Second, the authors note that although CPA is currently designed to predict the effects of perturbations on gene expression, the framework could be extended to incorporate additional types of single‐cell data, such as epigenomic, proteomic, or spatial measurements. CPA predictions can also guide the design of perturbation experiments by nominating drugs or gene knockout or activation combinations that are predicted to have the most interesting or desirable effects. Finally, CPA holds promise for developing better therapeutics by predicting optimal drug combinations or personalized treatments.

## Disclosure and competing interest statement

JW has no competing interests to declare.
